# Mechanisms underlying therapeutic resistance of tyrosine kinase inhibitors in chronic myeloid leukemia

**DOI:** 10.7150/ijbs.86305

**Published:** 2024-01-01

**Authors:** Jingnan Sun, Ruiping Hu, Mengyuan Han, Yehui Tan, Mengqing Xie, Sujun Gao, Ji-Fan Hu

**Affiliations:** 1 Hematology Department, First hospital of Jilin University, Changchun, Jilin, 130021, P.R. China.; 2 Stanford University Medical School, Palo Alto Veterans Institute for Research, Palo Alto, CA94304, USA.; 3 Oncology Department, Cancer hospital Chinese Academy of Medical Sciences, Langfang District, 065001, P.R. China.

**Keywords:** chronic myeloid leukemia, TKI Resistance, ABL mutation, mechanism

## Abstract

Chronic myeloid leukemia (CML) is a malignant clonal disease involving hematopoietic stem cells that is characterized by myeloid cell proliferation in bone marrow and peripheral blood, and the presence of the Philadelphia (Ph) chromosome with BCR-ABL fusion gene. Treatment of CML has dramatically improved since the advent of tyrosine kinase inhibitors (TKI). However, there are a small subset of CML patients who develop resistance to TKI. Mutations in the ABL kinase domain (KD) are currently recognized as the leading cause of TKI resistance in CML. In this review, we discuss the concept of resistance and summarize recent advances exploring the mechanisms underlying CML resistance. Overcoming TKI resistance appears to be the most successful approach to reduce the burden of leukemia and enhance cures for CML. Advances in new strategies to combat drug resistance may rapidly change the management of TKI-resistant CML and expand the prospects for available therapies.

## Introduction

Chronic myeloid leukemia is a myeloproliferative disease that originates from pluripotent stem cells carrying a characteristic reciprocal translocation between an Abelson leukemia virus (ABL) oncogene from the long arm of chromosome 9 and the breakpoint cluster region (BCR) from the long arm of chromosome 22. The fusion protein encoded by the BCR-ABL fusion gene has abnormally tyrosine kinase activity, leading to increased cell proliferation through its downstream signaling pathways. As the cause of CML, the resulting BCR-ABL fusion protein, is an ideal therapeutic target. The emergence of first and second-generation tyrosine kinase inhibitors (TKI) has led to overall survival (OS) rate of 82%-95% for CML. However, about 20%-30% of the patients ultimately develop resistance to TKI^1^. Here, we summarize recent advances on the mechanism of TKI resistance and the characteristics of mutations in the ABL kinase domain.

## Epidemiology

The incidence of CML is (1.6-2)/100,000 worldwide, accounting for 15% of all patients with leukemia^2^. In Western countries, patients older than 70 years comprise more than 20% of CML patients, while children and adolescents account for < 5% of all cases, with the median age of CML patients about 57 years^1^ . In Asia and Africa, the median age at diagnosis is less than 50 years^1^. At the end of the 1990s, imatinib mesylate (IM) was successfully used to treat CML, ushering in the era of molecularly targeted cancer therapy. According to the International Collaborative Research Group, the overall survival rate of IM treatment at 5 years is 90%-95%, and the Progression-free survival (PFS) at 5 years is nearly 80%-90%^3^, ^4^. At 10 years, the OS is stil 82%-85%, but the resistance rate to IM in first-line patients is 10%-15%, and the resistance rate to second-generation TKI is < 10%^5^. Summarizing multiple studies worldwide, mutations in the ABL kinase domain accounts for about 22.4%-54.46% of resistance CML patients, with p-loop mutations accounting for about one-fourth^6^, ^7^.

## Definition of TKI resistance

Primary resistance refers to the lack of hematologic, cytogenetic, or molecular response to TKI in the early stages of treatment. Secondary resistance refers to the loss of response after a patient has gained a certain degree of therapeutic response^8^.

The latest European Leukemia Network (ELN) treatment guidelines and the National Comprehensive Cancer Network (NCCN) CML Clinical Practice Guidelines consider clinical responses to include "best", "failure", and "warning"^1^, ^9^. TKI resistance is defined as "failure" in the evaluation of CML treatment response, referring to the definition of ELN failure in 2020 recommendations, with BCR-ABL^IS^ > 10% if confirmed within 1-3 months, BCR-ABL^IS^ > 10% at 6 months of TKI treatment, BCR -ABL^IS^ > 1% at 12 months of treatment, or BCR-ABL^IS^ >1% at any time after 12 months of treatment, with resistance mutations, the emergence of high-risk additional chromosome abnormalities (ACA)^10^, ^11^. The same definitions are recommended for second-line treatment^1^.

BCR-ABL fusion genes are necessary for CML to develop; however, the BCR-ABL oncogene alone is not sufficient to explain disease progression^11^, ^12^. In fact, BCR-ABL transcript levels increase with disease progression, promoting a secondary molecular, chromosomal-level hit and ultimately leading to the expansion of malignant cell clones^13^. Once a second strike is obtained, TKI therapy that inhibits BCR-ABL alone tends to fail^14^. Although the ultimate source of disease progression from these additional strikes remains BCR-ABL, it also suggests that the causes of disease progression include pathways other than ABL-dependent mechanisms.

## Mechanisms of TKI resistance

**Figure 1:**
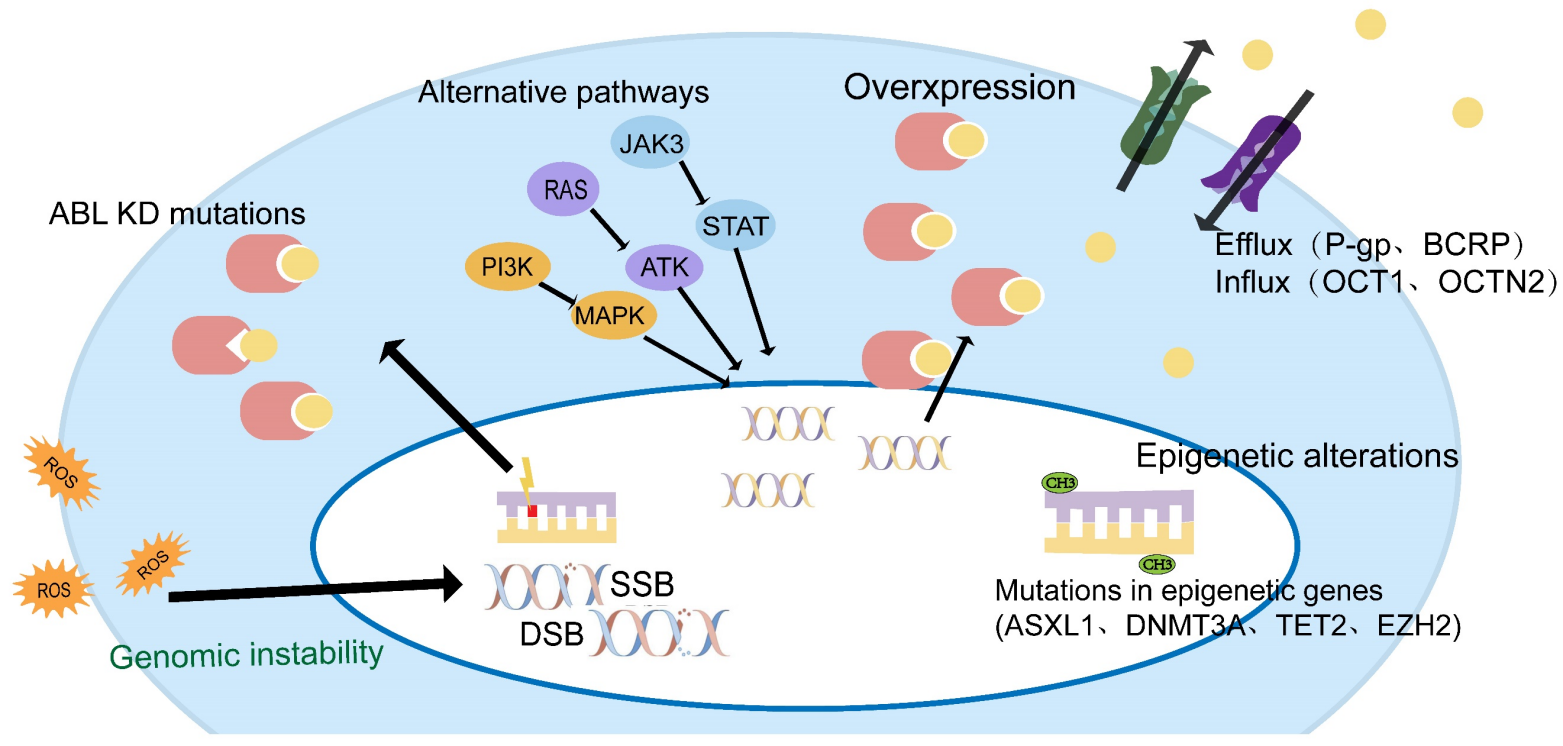
Mechanisms of TKI resistance in CML patients.

TKI resistance can be driven by ABL-dependent and independent mechanisms, depending on whether they are associated with the ABL kinase domain. Both mechanisms can induce significant clinical resistance, but secondary resistance usually involves ABL-dependent pathways, such as BCR-ABL mutations, gene amplification, or increased expression^15^, ^16^. ABL-independent resistance is more commonly seen in primary resistance, such as genomically unstable, quiescence leukemia stem cells, or individual differences in IM blood concentrations due to differences in oral bioavailability, the high affinity of serum proteins for IM, and cellular influx/efflux transporters^14^, ^17^, ^18^ (Figure 1).

CML-chronic phase (CMP-CP) progression to CML-blastic phase (CML-BP) is a multifactorial, multi-step process. It is believed that disease progression may be triggered by a series of different but equivalent events^14^. ABL-dependent pathways and non-dependent pathways may work in synergy, leading to the accumulation of key events at the DNA, RNA, and protein levels, causing abnormal cell cycle control, differentiation, apoptosis failure, and eventually drug resistance.

### ABL-dependent mechanism

#### BCR-ABL kinase domain mutations

**Figure 2:**
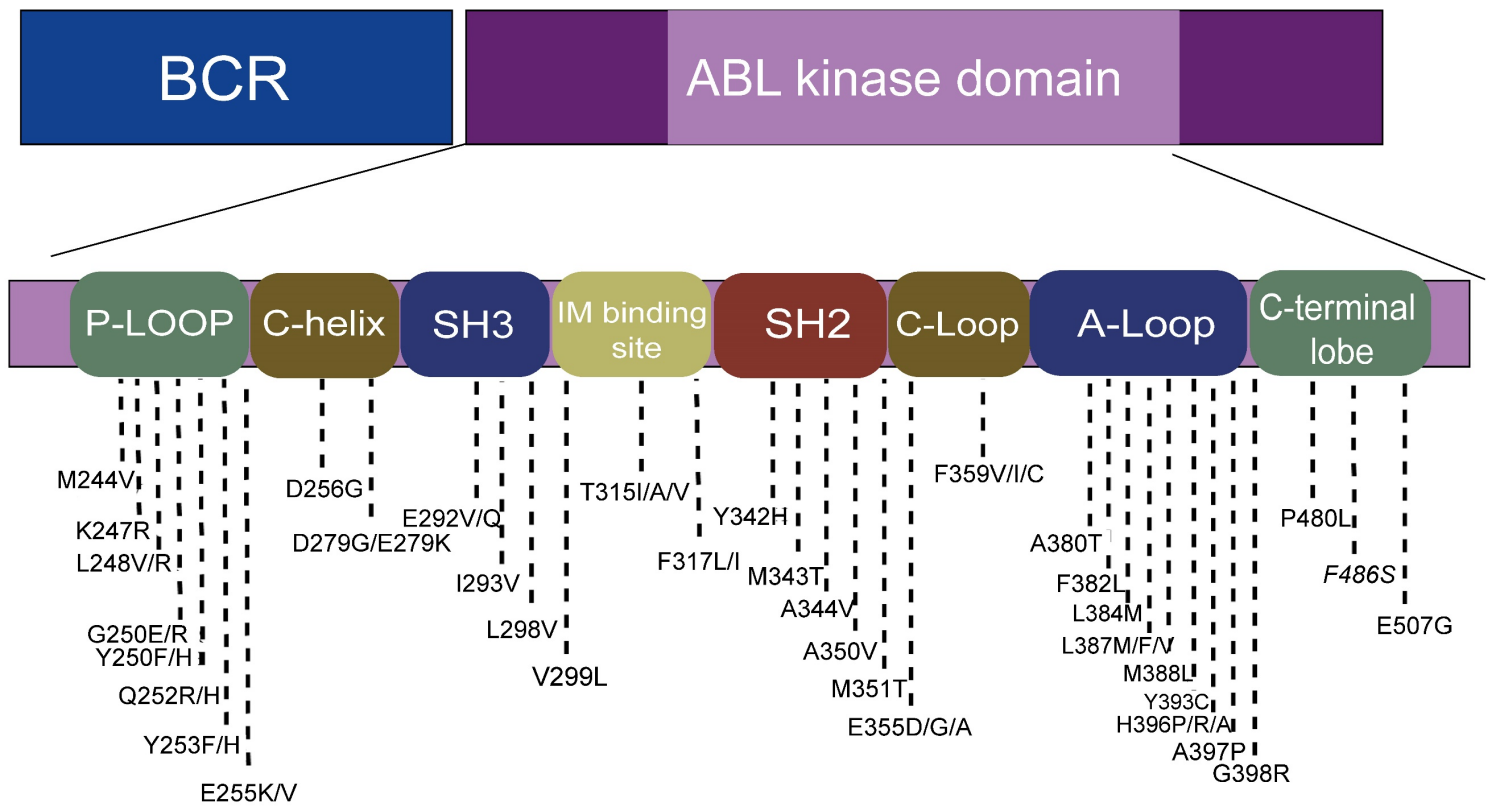
The location of hotspot mutations in the kinase domain.

Point mutations in the BCR-ABL fusion gene are the most common cause of TKI resistance, with about 31%-63% of imatinib (IM) resistance attributed to point mutations^19^-^21^. According to the published literature, more than 100 mutations have been found, distributed in the ABL amino acid range of 220-500 (Figure 2)^19^. Four main point mutation sites leading to IM resistance are: a). Gatekeepers (IM-binding site): F317I/L and T315I; b). ATP-binding loop (P-loop): E255K, Q252H, Y253F; c). catalytic-loop (C-loop): M351T; and d). activation loop (A-loop): H396P. The T315I mutation was the most common mutation (about 13%-16%). E255K and M351T mutations accounted for 9%-14% of mutations. Y253F, Q252H, F317L and other mutations accounted for 3%-6% of mutations, while V299L mutations were rare^6^, ^16^, ^22^. It is worth noting that E255K, Y253F, and Q252H, the mutations with higher mutation rates, are located in the p-loop, followed by the mutations at IM-binding sites^23^, ^24^.

Compound mutations (variants containing ≥ 2 mutations within the same BCR-ABL1 allele that presumably arise sequentially or simultaneously) exhibit different spectra of resistance from single mutations^10^. For example, while ponatinib is effective in treating a CML with a single T315I mutation, most compound mutations containing T315I are resistant to ponatinib^25^, ^26^. In addition to T315I, there are also E255V mutations that contain second mutations that exhibit different resistance to ponatinib. Meanwhile, compound mutations are mostly located in the p-loop in patients with CML, yet more likely to contain T315I^27^. Patients with compound mutations are more likely to progress during follow-up 28, 29.

Both ELN and NCCN recommend screening for BCR-ABL kinase domain mutations and providing the best drug regimen for a subset of well-documented mutation types, but there may not be definite TKI options for mutations listed in official guidelines.

Heat maps are widely used in the literature, which use the same reds, greens, and yellows as traffic lights to indicate the sensitivity of mutations to different TKI, allowing the selection of appropriate TKI for treatment^6^. It should be noted that heat maps are mutant models based on mouse cell lines and *in vitro* experiments. The sensitivity of *in vitro* generated data to TKI may vary *in vivo*. Similarly, there may be genetic differences between humans and mice. In addition, the choice of using heat maps to predict TKI is based on resistance caused by KD mutations as a prerequisite. If the resistance is driven by other cells or other pathways and is not related to KD mutations, the use of heat maps to predict TKI response is ineffective^15^. Even so, heat maps can still be used as a reference, but at the same time *in vivo* responses should be monitored to assess the biological behavior of subclonals.

Historically, Sanger sequencing (SS) has been used in most clinical practice to identify BCR-ABL1 mutations associated with TKI resistance. However, SS is unable to detect mutations present in 10% to 20% cells and does not allow direct detection of compound mutations, although their presence can be inferred in certain cases (when ≥ 2 mutations are detected at a combined frequency > 100%)^29^-^31^. Next‐generation sequencin (NGS) also enables direct detection of BCR-ABL1 compound mutations. However, it has recently been shown that polymerase chain reaction (PCR) mediated recombination may cause compound mutation frequencies to be overestimated when PCR amplicons are used for NGS^32^. Thus, the ability of NGS to reliably detect compound mutations, and therefore their prevalence in CML, remains to be established. NGS can be used to better monitor the size of the subclonal KD mutant and adjust the treatment regimen in time. Although SS cannot distinguish between polyclonal and compound mutations, unless the combined mutant allele burden clearly exceeds 100%, NGS identifies compound mutations as long as the average read length exceeds the distance between the 2 single nucleotide variations^30^, ^33^.

#### BCR-ABL overexpression

BCR-ABL overexpression is another mechanism of ABL-dependent resistance that causes IM resistance, but its clinical significance for resistance is far less than that of ABL mutation. BCR-ABL overexpression refers to any abnormality of the regulatory mechanism of ABL genes. Differential regulation or gene amplification can lead to increased expression of BCR-ABL^34^. Therefore, higher level of BCR-ABL proteins can still cause disease progression despite the administration of TKI. Compared with CML cells with low BCR-ABL expression levels in the chronic phase, high-expression cells are less sensitive to IM. The high expression of BCR-ABL is more pronounced in the accelerated phase (AP). It may be the reason why patients in accelerated phase treated with IM respond less well than patients in chronic phase.

#### DNA damage repair and Gene instability

Genomic instability leads to the accumulation of mutations at the ABL KD and other molecular or chromosomal aberrations. Vice versa, overexpression of BCR-ABL fusion proteins can also lead to genomic instability in CML cells^20^. Partial deletions of *RUNX1* and *PMRD16*, expression of *RUNX1/PMRD16*, and mutations in *GATA2* activation are also associated with CML progression and their presence may be detected in CML^14^, ^35^.

Abnormally active tyrosine kinase activity causes the accumulation of reactive oxygen species (ROS). Increased ROS can damage DNA, leading to alkaline oxidative damage, DNA double-strand breaks (DSBs), and mismatch repair. Ultimately, ROS-induced genomic instability and subsequent genetic events, such as mutations, chromosomal translocations, and deletions, can lead to drug resistance^24^, ^36^. ROS involvement in genomic instability and CML progression has been widely evaluated. BCR-ABL kinase activity has been found to increase intracellular ROS levels, which is significantly more pronounced in CML-BP cells, exhibiting higher BCR-ABL levels than in CP CML cells^19^. Elevated ROS levels and exogenous factors, such as radiation or genotoxic compounds, may enhance oxidative DNA damage. On the other hand, DNA repair mechanisms are dysregulated due to the loss or acquisition of BCR-ABL-positive cell function. In human cells, DSBs are preferentially repaired by homologous recombination (HR) or non-homologous termination (NHEJ), but sometimes highly unfaithful single-strand annealing (SSA) mechanisms may occur^37^. Novicki et al. have demonstrated that HR and NHEJ are enhanced in ROS-mediated DSB repair in BCR-ABL cells, where these mechanisms lead to mutations and a large number of deletions. In fact, BCR-ABL (non-mutant and T315I mutants) has been shown to bind and phosphorylate RAD51 and its paralog RAD51B, promoting unfaithful homologous HR in a dose-dependent manner^20^.

### ABL-independent mechanism

#### Alternative pathways

TKI inhibits the BCR-ABL kinase activity through competitive binding, but does not eliminate CML cells. This means that CML stem cells can survive through other signaling pathways, such as SRC, JAK/STAT, RAS/MAPK, and PI3K/AKT^38^. Activation of alternative pathways may reveal why the disease still recurs after patients who achieve an excellent response stop treatment^14^, ^39^, ^40^.

Overexpression of SRC family kinase proteins, such as LYN and HCK, is essential for cell proliferation, survival, and adhesion^19^, ^20^. SRC proteins lead to AKT activation and promote survival and STAT5 activation to stimulate proliferation. Overexpression of SRC proteins in CML is a rationale for the development and use of dual SRC/ABL inhibitors, such as dasatinib and bosutinib^35^.

In addition, STAT can activate the JAK2 protein by responding to cytokines released by cancer cells and bone marrow niche cells. JAK2 is activated and subsequently phosphorylated by one of the 7 STAT members. STAT3 and STAT5 have been identified as the most relevant STAT proteins in cancer^36^, ^41^. After STAT phosphorylation, this protein migrates to the nucleus, where it regulates transcription of various target genes, such as c-MYC.

GAB2 is a member of the GAB family of docking proteins that play a key role in CML by amplifying BCR-ABL signaling. Dysregulation of this protein leads to increased proliferation, decreased demand for growth factors, and increased cell viability^20^. In addition, continuous phosphorylation of GAB2 leads to activation of substrates, such as the stabilization of RAS proteins in active form after GAB2 activation. An increase in protein kinase C (PKC) expression has also been observed in TKI-resistant CML cells^39^.

After PI3K is activated, AKT is subsequently phosphorylated, affecting a variety of downstream proteins^42^. BAD is one of the AKT targets that reduces the signal of apoptosis. After phosphorylation, BAD becomes inactive and therefore does not inhibit anti-apoptotic proteins, such as BCL-2 and BCL-XL. Another AKT target is the FOXO transcription factor, which regulates cell cycle arrest and apoptosis under normal conditions. AKT-induced FOXO phosphorylation blocks its activity, and avoids apoptosis and promotes cell cycle progression. In addition, mTOR is a serine/threonine kinase that is activated by AKT and regulates mRNA translation, controlling cell growth and proliferation. Similarly, NF-kB is also indirectly activated by AKT, promoting gene transcription. AKT targets IKK, a natural inhibitor of NF-kB, and releases this inhibitory signal from NF-kB^34^, ^38^, ^43^

#### Quiescent CML stem cells

CML stem cells account for about 0.5% of the CD34+ population, and they do not require BCR-ABL kinase activity to survive^42^. CML stem cells are resistant to TKI, and resistance/recurrence is presumed to come primarily from these cells. *In vitro* studies have shown that "quiescent" leukemia stem cells are highly resistant to IM. Even if complete molecular biological remission is obtained, leukemia stem cells in some patients can still survive for a long time^34^, ^43^. This could explain why TKI cannot kill all leukemia cells even in patients with optimal response. So far, combining TKI with another drug to remove residual stem cells and identifying the underlying signaling pathways of CML stem cells seems to be the most promising way to overcome treatment failure^44^.

#### Epigenetic alterations

There is now ample evidence that mutations in epigenetic regulatory genes, such as DNMT3A, TET2, EZH2, and ASXL1, are relatively rare in chronic-phase CML^22^, ^45^, but the incidence of these mutations increases during disease progression and accelerates leukemia stem cell production, maintenance, and progression of CML^35^, ^46^, ^47^.

The most common mechanisms of epigenetic modification include methylation, acetylation, and phosphorylation^35^. Their role is to regulate chromatin structure and remodeling, providing a site for the recruitment of other transcription factors, followed by altering the cell cycle, apoptosis, and expression of tumor suppressor genes. DNA hypermethylation is a common carcinogenic process in many solid and hematological tumors^48^. It has been reported in detail in patients with CML, especially in patients with AP and BC. Although ABL hypermethylation has been demonstrated, its role in the pathophysiology of disease progression is unclear.

#### Bioavailability and blood concentration

As an oral drug, IM is first affected by patient compliance, followed by IM absorption through the gastrointestinal tract, and is influenced by first-pass metabolism. About 95% of IM binds to plasma proteins (mainly albumin) and α-1 acid glycoprotein (AGP, a hepatic acute phase protein)^49^. It has been proposed that the combination of AGP with IM in plasma can reduce the accessibility^34^, ^35^, ^50^.

TKIs (imatinib, nilotinib, dasatinib) are metabolized primarily by the cytochrome P450 system, mainly involving the isoenzyme CYP3A4. The activity of this isoenzyme varies from individual to individual and is affected by concomitant drugs, which may also lead to differences in IM concentrations.

#### Drug influx/efflux pump

Resistance is related to the expression level and function of solute carrier (SLC) transporters. According to the direction of transportation, it is divided into influx type and efflux type transporter. Influx transporters include organic cation transporter 1 (OCT1 or SLC22A1), organic anion-transporting polypeptide 1A2 (SCL01A2 or OATP1A2), OCTN2 and MATE1^46^, ^51^. OCT1 is the main transporter responsible for TKI uptake, and its expression or activity affects the level of drug response^7^, ^13^, ^27^. Other transporters have been identified as intermediaries in TKI transportation.

Efflux transporters include ATP-binding cassette subfamily B member 1 (ABCB1) also known as P-glycoprotein or MDR1, and ATP-binding cassette G subfamily member (ABCG2) also known as breast cancer resistance protein (BCRP)^52^. All TKIs approved for CML therapy are recognized P-gp substrates, and high levels of ABCB1 expression (genes encoding P-gp) are associated with poorer long-term outcomes and advanced disease. Another basic transporter of TKI drug resistance is breast cancer resistance protein (BCRP), encoded by the ABCG2 gene^35^. This protein is found in stem cells and its function is particularly relevant to leukemia stem cells (LSCs), protecting them from TKI action.

Reduced *ABCG2* and increased *SLC22A1* mRNA expression are associated with imatinib response in chronic myeloid leukemia. High expression of ABCB1 is more likely to be observed in patients with acute phase CML than in patients with CML in the chronic phase^47^. In patients undergoing IM therapy, higher OCT-1 activity was associated with increased MMR, EFS, and OS, while cell uptake of second-generation TKIs (dasatinib and nilotinib) appeared to be independent of OCT expression, thus supporting the theory that dasatinib or nilotinib might be superior^19^, ^39^, ^50^.

## Innovative strategies

Currently approved TKIs mainly target the ATP binding site of BCR-ABL1. Asciminib (ABL001) is a potent, specific, orally bioavailable BCR-ABL1 inhibitor that is distinct from approved ABL1 kinase inhibitors in that it does not bind to the ATP-binding site of the kinase^53^. In contrast, asciminib acts as an allosteric inhibitor and engages a vacant pocket at a site of the kinase domain normally occupied by the myristoylated N-terminal of ABL1— a motif that serves as an allosteric negative regulatory element lost on fusion of ABL1 to BCR^2^. By binding the myristoyl site, asciminib mimics myristate and restores inhibition of kinase activity. Owing to the distinct conformation of the myristoyl pocket, asciminib has high selectivity for ABL1 ABL kinase mutations, including T315I^11^, ^15^. Asciminib targets both native and mutated BCR-ABL1, including the gatekeeper T315I mutant.

## Conclusion

The prognosis of CML has been significantly improved since the discovery of molecularly targeted therapies, but due to the heterogeneity of the mechanisms of resistance, the focus is shifting to find an inhibitor with broad utility that is conducive to overcoming drug resistance. Therefore, the management of patients with drug-resistant CML, including next-generation treatment options and higher sensitivity monitoring techniques, remains a challenge.
